# Kinetics of Microwave-Assisted Extraction Process Applied on Recovery of Peppermint Polyphenols: Experiments and Modeling

**DOI:** 10.3390/plants12061391

**Published:** 2023-03-21

**Authors:** Branimir Pavlić, Muammer Kaplan, Zoran Zeković, Oltan Canli, Nebojša Jovičić, Danijela Bursać Kovačević, Anica Bebek Markovinović, Predrag Putnik, Oskar Bera

**Affiliations:** 1 Faculty of Technology, University of Novi Sad, Bulevar Cara Lazara 1, 21000 Novi Sad, Serbia; bpavlic@uns.ac.rs (B.P.);; 2 TUBITAK Marmara Research Centre, Institute of Chemical Technology, P.O. Box 21, Gebze 41470, Kocaeli, Turkey; 3 TUBITAK Marmara Research Centre, Environment and Cleaner Production Institute, P.O. Box 21, Gebze 41470, Kocaeli, Turkey; 4 Faculty of Food Technology and Biotechnology, University of Zagreb, Pierottijeva 6, 10000 Zagreb, Croatia; 5 Department of Food Technology, University North, Trg dr. Žarka Dolinara 1, 48000 Koprivnica, Croatia

**Keywords:** *Mentha piperita* L., microwave-assisted extraction (MAE), kinetics, modeling, polyphenols

## Abstract

The aim of this work was to investigate the microwave-assisted extraction (MAE) kinetics of polyphenolic compounds from organic peppermint leaves. The phytochemicals of peppermint (*Mentha piperita* L.) are increasingly used in food technology due to their numerous biological activities. The processing of various plant materials by MAE and the production of high-quality extracts is becoming increasingly important. Therefore, the influence of microwave irradiation power (90, 180, 360, 600, and 800 W) on total extraction yield (Y), total polyphenols yield (TP), and flavonoid yield (TF) were investigated. Common empirical models (first-order, Peleg’s hyperbolic, Elovich’s logarithmic, and power-law model) were applied to the extraction process. The first-order kinetics model provided the best agreement with the experimental results in terms of statistical parameters (SS_er_, *R*^2^, and AARD). Therefore, the influences of irradiation power on the adjustable model parameters (*k* and *C_eq_*) were investigated. It was found that irradiation power exerted a significant influence on *k*, while its influence on the asymptotic value of the response was negligible. The highest experimentally determined *k* (2.28 min^−1^) was obtained at an irradiation power of 600 W, while the optimal irradiation power determined by the maximum fitting curve determination predicted the highest *k* (2.36 min^−1^) at 665 W.

## 1. Introduction

Medicinal plants and pharmaceutical formulations derived from natural sources are currently gaining interest as substitutes for synthetic drugs. The main reasons for this are certain limitations of conventional medicines, e.g., side effects, misuses, and negative impacts of the chemical industry on the environment. Therefore, a large part of our population is turning to traditional medicine and natural products. However, the raw materials used for obtaining bioactive compounds, such as medicinal/aromatic plants, are not always adequately utilized by the modern food, cosmetic, and pharmaceutical industries since the conventional extraction approach does not necessarily provide satisfactory results in terms of yield of bioactive compounds and quality of extracts. The unsuitable uses of these resources are usually associated with the applications of traditional extraction techniques, which are not the best fit for the economic aspects of the production processes, such as the consumption of raw materials, and expenditures of time and energy [1]. Generally, extraction techniques have been modified to reduce the consumption of resources and improve the quality and yield of the final products [2]. For this purpose, various emerging extraction methods deploy utilization of ultrasounds, pulsed electric fields, microwaves (MAE), pressurized liquids, and supercritical fluids in order to accelerate and improve extraction process [3,4,5,6]. The main goal in developing novel extraction techniques is the ability to achieve high process efficiency, and high yields from sustainable resources without the consumption of toxic chemicals and solvents [7]. These techniques should fulfill the main aspects of “green” chemistry: use of alternative solvents, reduction of product purification steps, recovery of co-products from waste streams, and production of biodegradable extracts without the presence of contaminants [8].

The main issues associated with common valorization approach are limitations of the traditional extractions, which restrict economic aspects of production in terms of energy and resource preservation as well as yields of valuable extracts [9]. These are the main reasons for the recent shift in focus towards research, development, and application of alternative extraction techniques.

Microwave-assisted extraction (MAE) has been widely used to extract bioactive compounds from natural resources. Non-ionizing electromagnetic waves with a frequency of 0.3–300 GHz are responsible for the acceleration of the mass transfer in the MAE processes. This occurs due to interaction of microwaves and polar molecules, which leads to heat generation in the extractor [10]. Ionic conduction and dipole rotation are the main mechanisms of heat generation and absorption of microwaves depends on the polarity of absorbing molecules, i.e., their dielectric constant [4]. Moreover, the gradient of heat and mass transfer in MAE is from the solid to the liquid phase, in contrast to conventionally heated extraction, where the gradient of heat transfer is from the liquid to the solid phase [11]. Due to its properties, MAE has recently been used for the recovery of various bioactive compounds such as polyphenols, quinones, terpenes, and alkaloids [12]. Most novel approaches, including MAE, have proven their effectiveness in addressing these challenges with significant improvements over conventional methods, as the extracts provided high yields of target compounds and the “green” nature of the process is maintained.

Peppermint (*Mentha piperita* L.) is well known as an important industrial plant because of its medicinal, aromatic, and culinary properties. Furthermore, the dried peppermint leaf (*Menthae piperitae folium*) and its essential oil (*Menthae piperitae aetheroleum*) are official pharmaceutical preparations according to the European Pharmacopoeia [13]. Peppermint has shown various biological activities, the most notable are antimicrobial and antioxidant effects which were reviewed elsewhere [14]. It has shown other therapeutic properties such as antitumor, antiallergic, and immunomodulatory effects and benefits to the digestive tract, etc., according to in vitro and clinical studies [15,16]. Terpenoids from the essential oil and polyphenols are considered to be the most important bioactives of peppermint. Therefore, it is important to develop new protocols for efficient extraction to improve the yield of these compounds. Moreover, organic production of medicinal plants is in line with the principles of green chemistry, as it recommends the use of renewable energy, the reduction of potential contamination, and the recycling of waste streams [17]. According to Ikiz and Demircan (2013), organic and conventional production of essential oil-bearing plants could differ in yield of the crop, cost of the production, and quality of the final product [18]. Organic crops, such as peppermint, are gaining more attention due to higher market value and better consumer acceptance compared to conventionally grown crops. 

Therefore, the aim of this work was to use organic peppermint as the raw material for the recovery of bioactive compounds (polyphenols and terpenes) using MAE. The extraction process was analyzed with focus on a kinetic study. The main aim of this work was to obtain different empirical kinetic models able to adequately describe the MAE process and to evaluate the effects of irradiation power on the yield of bioactive compounds, and on the model parameters.

## 2. Results and Discussion

**Figure 1:**
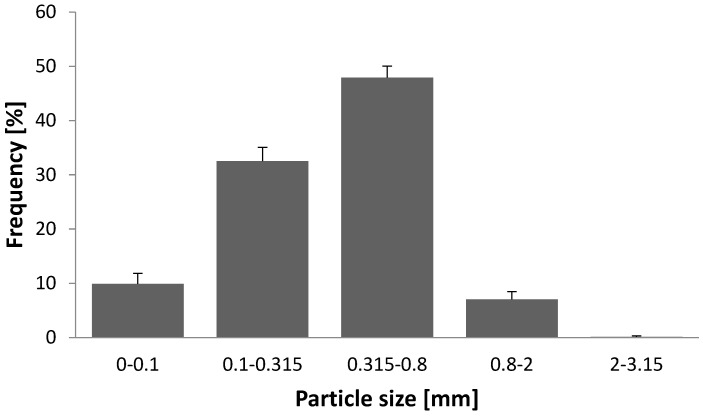
Particle size distribution of ground peppermint leaves.

Peppermint (*Mentha piperita* L.) leaves used as raw material for MAE were first characterized in terms of moisture content, essential oil content, and mean particle size and its distribution. The mean particle size of peppermint leaves was 0.224 mm, while their distribution is shown in Figure 1. Grinding of the plant material is considered an essential step in post-harvest processing because the mean particle size and distribution directly affect the mass transfer process. Ground peppermint leaves have a small particle size, which should limit mass transfer. However, the samples did not contain larger proportions (>10%) of very fine powder (>0.1 mm), which should prevent possible clogging of the filter material after solid–liquid extraction. The moisture content of the crude plant material was 8.77%, while the essential oil content was 2.325 ± 0.07%, indicating that the plant material was suitable according to the *European Pharmacopoeia* [8].

### 2.1. Extraction Kinetics

According to Dai et al. [19], MAE showed certain advantages in the extraction of bioactive compounds from peppermint leaves compared to conventional, reflux, and ultrasound-assisted extraction. Our previous study has shown that peppermint extracts obtained by MAE exhibit strong bioactive potential which is reflected in the potent in vitro antioxidant activity and enzyme-inhibitory potential in various model systems [20]. Therefore, MAE was chosen as a suitable extraction method to determine the extraction kinetics of extractable compounds. It is known that the efficiency of the extraction process is significantly affected by several parameters. In the case of MAE, solvent polarity and concentration, sample properties, sample-to-solvent ratio, irradiation power, temperature, extraction time, and stirring are considered to be the most influential process variables [21]. In this work, the influence of irradiation power on the extraction kinetics of Y, TP, and TF was investigated.

**Figure 2:**
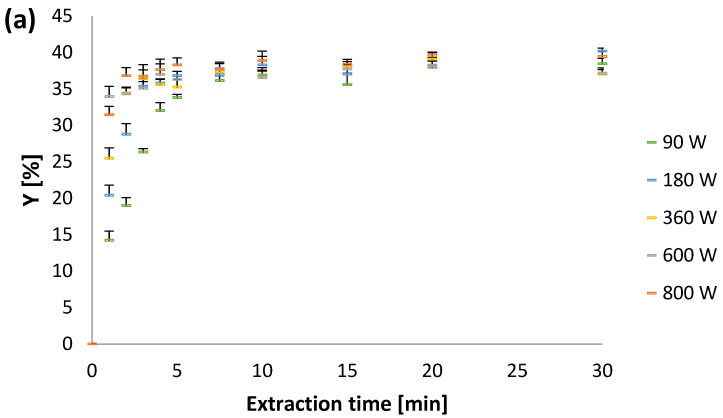

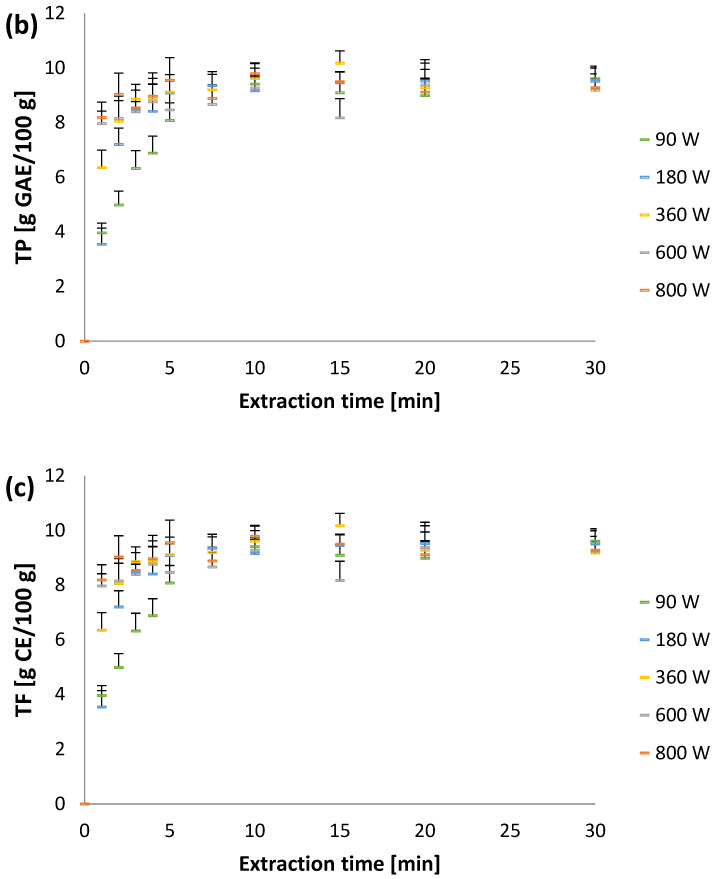
Experimentally observed values for: (**a**) Y, (**b**) TP, and (**c**) TF vs. extraction time.

After 30 min of extraction, Y varied from 37.02 to 40.17% (Figure 2a). The highest Y value was obtained at an irradiation power of 180 W. At the end of the process, no significant differences for Y were observed, because the extraction curve was far into the stationary phase after 30 min. The experimentally determined values of TP were 9.1731–9.6110 g GAE/100 g and the highest yield at the end of the extraction process was obtained with an irradiation power of 90 W (Figure 2b). According to Petkova et al. [8], the yield of TP obtained by MAE of peppermint was 37.7 ± 0.5 mg GAE/g, where MAE exhibited some improvement in polyphenol yield compared to conventional extraction. 

Additionally, Lv et al. [15] reported that TF in conventional and organic peppermint leaf extracts was 191.8 ± 10.2 and 190.9 ± 0.3 mg GAE/g. Furthermore, TP in peppermint leaf extracts was 9.67 ± 0.62 g GAE/100 g [22], which is in accordance with the data of this work. However, it could be concluded that the yield of TP may range widely due to the origins of the plant material, geographical locations, cultivation methods, and harvesting and storage conditions. According to our previous study [23], flavonoids (epicatechins, naringenin, eriodictyol, kaempferol, quercetin, rutin, isorhamnetin and luteolin, phenolic acids (vanillic acid, protocatechuic acid, caffeoyltartaric acid, fertaric acid, caffeic acid, *p*-coumaric acid, and coutaric acid), and stilbenes (*trans*-piceatannol and trans-resveratrol) were the major polyphenolic compounds identified in peppermint extracts obtained by MAE. The main identified flavonoid subgroups were flavan-3-ols, flavanones, flavonols, and flavones. Flavonols were either free or in form of their respective 3-*O*-gycosides with glucose, galactose, and rutinose as the carbohydrate compounds, while flavanones such as naringenin were free or in form of their 7-*O*-glucoside.

This was in accordance with the study of Riachi and Maria [16], where it has been reported that 53% of the constituents of TP are flavonoids, followed by phenolic acids (42%), and lignans and stilbenes (2.5%). The same authors reported that the predominant compounds were eriocitrin, rosmarinic acid, eriodictyol-glycopyranosyl-rhamnopyranoside, and luteolin 7-*O*-rutinoside. Further emphasis should be put on the potential utilization of peppermint by-products, such as post-hydrodistillation waste, since it has been confirmed that products derived could be used in food products [24].

The contents of TF in peppermint extracts ranged from 9.5881 to 10.3584 g CE/100 g, which is rather unexpected since flavonoids are the subgroup of polyphenols. However, it should be kept in mind that the spectrophotometric techniques used are often not very selective and certain compounds may interfere with the target compounds. According to Petkova et al. [25], the TF yield in the peppermint extract obtained by MAE was lower, i.e., 37.7 ± 0.5 mg/g.

It was observed that the initial extraction rate increased with the increase in irradiation power up to 600 W (Figure 2). However, further increasing the power to 800 W did not result in an additional increase in the initial slope. In addition, rapid extraction took up to 5 min, suggesting that the majority of all extractable compounds were readily eluted from the plant material. After that, internal diffusion extraction was performed, indicating that the extraction yield did not increase significantly with increasing extraction time. On the other hand, some decrease in Y and TP were observed after 15 min of extraction, which could be explained by the degradation of certain compounds. The degradation of bioactive compounds during MAE could be due to the release of excess energy generated by the microwaves and used as activation energy for various degradation reactions [10]. A similar trend of experimental data was observed for Y, TP, and TF (Figure 2). However, the decrease in TP after 15 min of extraction was even more pronounced at irradiation powers of 360, 600, and 800 W (Figure 2b).

### 2.2. Mathematical Modeling

Obtained experimental data (Figure 2) were fitted by the four empirical models described with Equations (1) and (4)–(6) and adjustable parameters are presented in Table 1 and Table 2 for Y and TP, respectively. Due to the rather unexpected phenomenon of experimentally obtained TF having higher values than TP and certain similarities in kinetic curves for all three responses, mathematical modeling of TF curves was not performed. Accordance between experimental data and applied models was described by sum of squared errors (SS_er_), coefficient of determination (*R*^2^), and average absolute relative deviation (AARD) which are also given in Table 1 and Table 2. The mean *R*^2^ values for the first-order, Peleg’s hyperbolic, and power-law model were particularly high (>0.950), while the mean *R*^2^ value for Elovich’s logarithmic model was significantly lower (0.729). Furthermore, first-order and Peleg’s hyperbolic models had satisfactory SS_er_ (10.615 and 18.224) and AARD (2.52 and 3.35%), while mean values were significantly lower compared to those of Models III (51.644 and 5.71%) and IV (65.969 and 6.46%). Therefore, the first-order model provided the best fit with experimentally obtained values of Y. A similar situation occurred with the modeling of TP, since Model I also provided the best fit with the experimental data and the highest mean value for *R*^2^ = 0.984. Here, mean values for the SS_er_ (2.12) and AARD (3.84%) were the lowest compared to other models. In contrast, according to statistical parameters, Elovich’s and the power-law models provided slightly weaker fits with experimental data of TP (Table 2). Additionally, it can be noted that SS_er_ values were lower when the irradiation power was high, especially for Elovich’s and power-law models. The reason for this is that the maximum TP concentration was achieved much faster at high irradiation power and the curve becomes horizontal line after a short time. This causes the average vertical distance between the experimental points and fitted curve to become smaller, and thus SS_er_ is decreasing.

**Figure 3:**
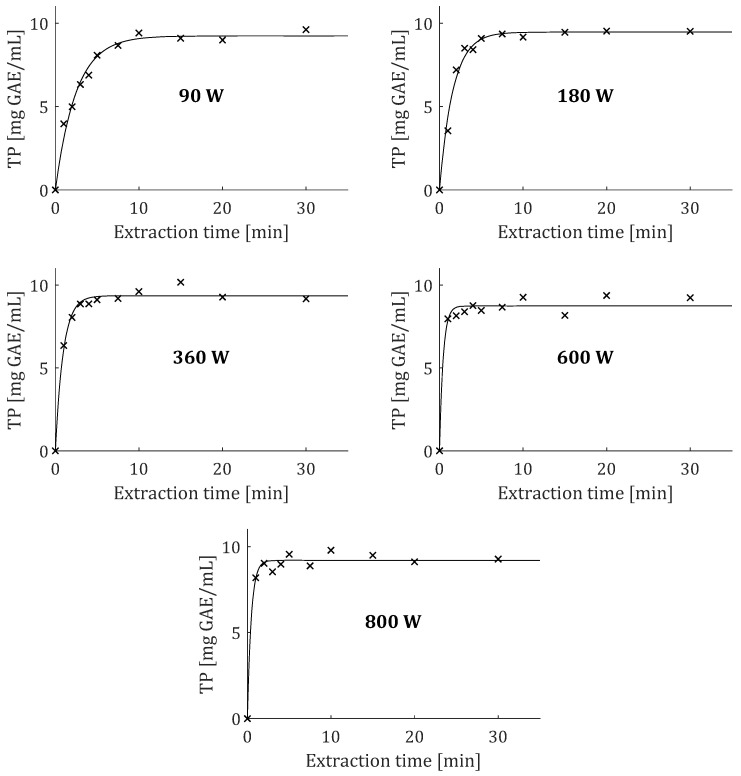
Influence of microwave irradiation power on fitting curves of TP for first-order model.

**Figure 4:**
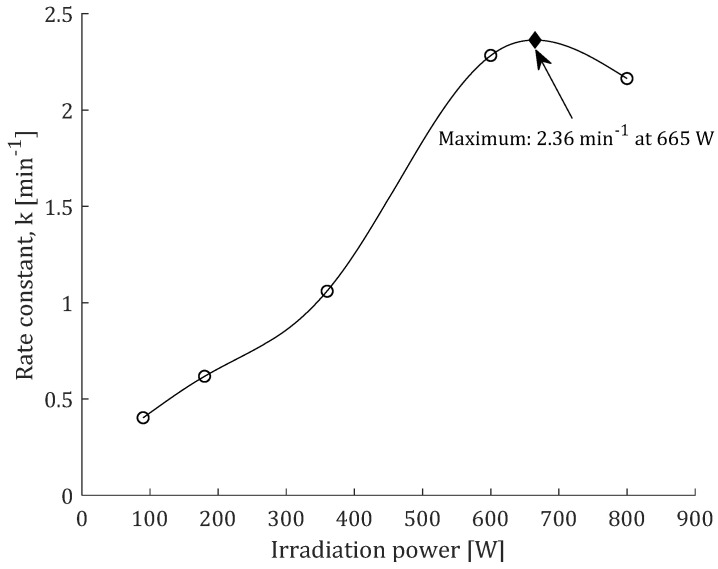
Influence of microwave irradiation power extraction rate constant (*k*_1_) calculated from Model I.

Because the first-order model provided the best fit for the experimental data for TP, its fitted curves obtained at different microwave irradiation power levels are presented in Figure 3. *C_t_* and *k*_1_ were adjustable parameters in the equation of the first-order model. According to the results in Table 2, it was observed that the irradiation power had a rather indeterminate effect on *C_t_*. Alternatively, *k*_1_ increased with the increase in irradiation power up to 600 W, after which *k*_1_ decreased with further increase in irradiation power up to 800 W (Figure 4). It was reported that the increase in irradiation power affected the extraction solvent and changed its polarity, viscosity, and surface tension, leading to an increased release of solutes from plant cells [26]. 

Furthermore, higher irradiation power accelerated molecular movement and internal diffusion, resulting in the degradation of the plant material [27]. This allowed easier penetration of the extraction solvent and reduced the mass transfer limits for internal diffusion from the solid to the liquid phase. However, higher irradiation power could lead to excessive energy absorption by the samples, possibly resulting in degradation of the target compounds. According to Mandal and Mandal [28], increasing the irradiation power increased the extraction yield up to a certain limit and then further increase becomes insignificant, which is consistent with the results of the present work (Figure 2). In addition, increasing the irradiation power in a similar experimental domain (400–800 W) did not significantly increase the TP yield in MAE of coriander seeds and basil leaves [29]. 

Irradiation power had a negative effect on TP in MAE of *Morus nigra* fruits; however, the effect was still smaller compared to other MAE parameters, such as ethanol concentration and extraction time [30]. The decrease in *k*_1_ at 800 W could be explained by the rapid denaturation of certain compounds at the beginning of the MAE process due to excessive energy and heating (Table 2), while a further increase in yield at later stages of extraction could be due to the release of bond polyphenols (Figure 2b). It can be concluded that the irradiation power exerts a significant influence on *k*_1_, while its influence on the asymptotic value of response could be neglected. Thus, the optimal irradiation power should provide the highest *k*_1_, which was experimentally achieved at 600 W (Figure 4). 

According to Dai et al. [19] increased extraction time in the treatment of MAE beyond 30 min resulted in decomposition of peppermint essential oil compounds. It should also be noted that too long exposure of the sample to microwaves could potentially lead to the degradation of some polyphenolic compounds such as flavonoids [31]. Rate constant (first-order model) dependence from irradiation power was shown by the existence of a maximum (Figure 4). The influence of irradiation power on *k*_1_ was fitted using the cubic spline model and the predicted maximum (2.36 min^−1^) has been roughly estimated at 665 W. Therefore, further experiments should be performed with a fixed irradiation power of 600 W, while the influence of other MAE factors should also be evaluated.

**Figure 5:**
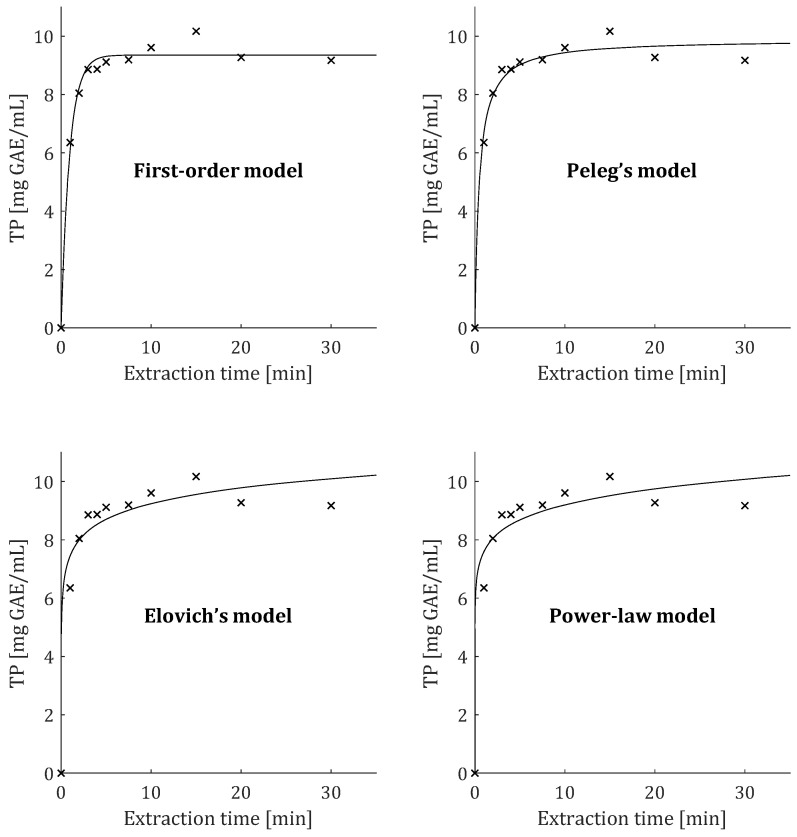
Experimental data and fitting curves of TP observed at 360 W for four kinetic models.

Fitting curves of four applied kinetic models and characteristic model parameters for TP observed in the sample which was obtained with 360 W of irradiation power are presented in Figure 5. The second-order hyperbolic model also provided an adequate fit with experimental data. Second-order rate constant ($k_{2}^{\prime }$) and saturation concentration (*C_eq_*) were calculated parameters in this model. It predicted slightly higher asymptotic values of Y and TP compared to Model I (Table 1 and Table 2). The power-law exponent (*n*) and extraction rate constant (*B*) were the adjustable parameters in the power-law model. Irradiation power exhibited a negative influence on the diffusion exponent, while the extraction rate constant increased at higher irradiation power in the case of both Y (Table 1) and TP (Table 2). 

The *B* constant provides information about system solvent–target compounds during MAE. Therefore, it could be concluded that irradiation power affected both solvent and sample properties. Since rapid extraction of target compounds occurs at the beginning of the process, the concentration gradient quickly drops which results in reduction in the diffusion exponent when higher irradiation power is applied. Parameter *a* from Elovich’s model is similar to *B* from the power-law model, which was confirmed by similar values of these parameters in the cases of Y and TP (Table 1 and Table 2). Irradiation power exhibited a positive influence on *a* and negative effects on *E*, suggesting that the extraction rate at the beginning of the process would be higher when higher irradiation power was applied. These empirical models provided certain information about investigated MAE systems; however, the first-order model was used for further calculations since it provided the best fit with experimental data.

### 2.3. Chemical Profile of Volatile Compounds

Microwaves have been previously used to accelerate hydrodistillation for the extraction of essential oils from *M. piperita* [32,33]. However, information about the chemical profile of peppermint volatiles in liquid extracts obtained by MAE is lacking. Since the highest experimentally observed *k* was obtained at irradiation power of 600 W, this sample was analyzed using GC-MS to determine the chemical profile of the volatile compounds. It was found that menthomenthene (29.32 mg/100 g), menthone (16.74 mg/100 g), 1,8-cineole (13.52 mg/100 g), d,l-limonene (10.65 mg/100 g), and *p*-cimene (11.36 mg/100 g) were the most abundant volatile compounds with >10 mg/100 g of plant material (Table 3). According to Orio et al. [32], monoterpenes were the most dominant compounds in peppermint essential oil obtained by microwave-assisted hydrodistillation (MWHD), followed by sesquiterpenes. It was observed that low molecular weight volatile compounds and monoterpenes were detected in the peppermint extract. This could be explained by the different mechanism of recovery in MAE and MWHD, as MWHD is focused towards recovery of volatile compounds. Volatile compounds obtained from peppermint have exhibited strong antioxidant and antimicrobial potential in meat products [34,35], suggesting that peppermint extracts obtained by MAE could be considered as natural additives in this type of foods.

Orio et al. [32] reported that menthol and menthone were the predominant constituents in *M. piperita* essential oils, followed by 1,8-cineole and limonene. Contrarily, Gavahian et al. [33] pointed out that neoiso-menthol, iso-menthone, and menthofuran were the major terpenoids in peppermint essential oils obtained by MWHD. Besides the influence of extraction techniques and applied conditions, the variation of chemical profile could be explained by geographical origin, influence of climate, and post-harvest processing. So, it is not surprising that carvone (67.9%) was the predominant compound in peppermint essential oil grown in Saudi Arabia, followed by limonene (10.4%), and 1,8-cineole (5.33%), as noted by Abdel-Hameed et al. [36]. This confirms the significant influence of above factors. Various menthane derivatives were detected in peppermint extract obtained by MAE: menthone, isomenthone, menthane, menthomenthene, L-(-)-menthol, and 5-methyl-2-(1-methylethyl)-2-cyclohexen-1-one (Table 3). This could be explained by potential chemical changes (oxidation, hydrolysis, etc.) that occur during MAE when an ethanol–water mixture is used as an extraction solvent [37]. Therefore, MAE of peppermint could be a suitable method for extraction of both polyphenols and volatile compounds from essential oils.

**Table 3:** Chemical profile of volatile compounds determined by GC-MS in *M. piperita* extract obtained at 600 W.

RT ^a^	Compound	RI_exp_–RI_lit_	Identification	Content [mg/100 g]
6.37	Acetic acid ethyl ester	615−609 ^c^	RI, MS	2.62
7.63	2-Methylbutanal	660−664 ^c^	RI, MS	0.44
8.87	Propanoic acid ethyl ester	710−716 ^c^	RI, MS	0.50
9.85	Isoamyl alcohol	748−756	RI, MS	0.67
11.88	Butanoic acid ethyl ester	810−804 ^c^	RI, MS	0.75
15.09	Styrene	-	MS	2.75
15.21	2,5-Diethyltetrahydrofuran	-	MS	0.21
15.27	Nonane	900−900 ^c^	RI, MS	6.94
16.18	Tricyclene	923−926 ^c^	RI, MS	0.35
16.51	α-Pinene	935−939 ^c^	RI, MS	0.55
16.72	α-Fenchene	949−952 ^c^	RI, MS	0.23
17.01	(+)-Camphene	-	MS	0.42
17.08	Camphene	958−954 ^c^	RI, MS	3.30
17.41	Benzaldehyde	969−960 ^c^	RI, MS	0.12
18.07	*p*-Menth-3-ene	985−987 ^c^	RI, MS	7.02
18.72	α-Phellandrene	1010−1002 ^c^	RI, MS	0.31
19.02	α-Terpinene	1018−1017 ^c^	RI, MS	4.98
19.24	*p*-Cymene	1025−1024 ^d^	RI, MS	11.36
19.38	d,l-limonene	1035−1029 ^d^	RI, MS	10.65
19.53	1,8-Cineole	1035−1031 ^c^	RI, MS	13.52
19.71	*cis*-Ocimene	1046−1037 ^c^	RI, MS	0.15
20.16	γ-Terpinene	1069−1059 ^c^	RI, MS	6.05
20.94	α-Terpinolene	1090−1088 ^c^	RI, MS	3.59
21.05	*p*-Cymenene	1095−1091 ^d^	RI, MS	1.66
21.21	2-Methyl butyl 2-methyl butyrate	1105−1100 ^c^	RI, MS	0.17
21.33	Amyl isovalerate	-	MS	0.49
22.86	Menthone	1148−1152 ^c^	RI, MS	16.74
23.11	Isomenthone	1160−1162 ^c^	RI, MS	7.88
23.18	Menthene	-	MS	0.45
23.26	5-Methyl-2-(1-methylethyl)-2-cyclohexen-1-one	-	MS	0.47
23.36	L-(-)-Menthol	1175−1171 ^c^	RI, MS	3.96
25.90	Menthomenthene	-	MS	29.32
26.35	3,7,7-Trimethyl-bicyclo [4.1.0]heptane	1295−1302 ^e^	RI, MS	0.59
28.11	β-Bourbonene	1381−1388 ^c^	RI, MS	0.23
∑TVC ^b^				150.70

RI_exp_, Kovat’s retention index calculated. RI_lit_, retention index reported in the literature. MS, comparison with mass spectra library; ^a^ Retention time, ^b^ Total identified volatile compounds; ^c^ Adams [38]; ^d^ Babushok et al. [39]; ^e^ Nikolaou et al. [40].

The results suggested that MAE provided coextraction of low molecular volatile compounds, mostly monoterpene hydrocarbons and their respective oxygenated derivatives. The presence of volatile terpenoids and polyphenols could improve bioactive potential of the peppermint extracts since both classes of bioactives are recognized as potent antioxidants. Antioxidant and antimicrobial activity should be further studied in order to provide in-depth information about peppermint extracts obtained by MAE which could be potentially used for the production of functional foods and dietary supplements.

## 3. Materials and Methods

### 3.1. Plant Material

Peppermint (*Mentha piperita* L.) was grown and harvested at the Institute of Field and Vegetable Crops (Novi Sad, Serbia) in 2015. The collected plant material (*Menthae piperitae folium*) was air dried, stored in paper bags, and kept at room temperature before the experiments, which were performed in 2019. The dried peppermint leaves were ground in a blender and the mean particle size and particle size distribution were determined using a sieve set (CISA Cedaceria Industrial, Barcelona, Spain). The moisture content of the plant material was determined gravimetrically by drying the samples at 105 °C until constant weight was achieved. Experiments were performed in triplicate and results are expressed as mean ± standard deviation.

### 3.2. Chemicals

1,1-Diphenyl-2-picryl-hydrazyl-hydrate (DPPH), (±)-catechin, and Folin–Ciocalteu reagent were purchased from Sigma (Sigma-Aldrich GmbH, Steinheim, Germany), while gallic acid and a standard mixture of n-alkanes (C7–C25) were obtained from Sigma-Aldrich (St. Louis, MO, USA). 1-Bromo-2-fluorobenzene was obtained from Absolute Standards, Inc. (Hamden, CT, USA). The ultra-pure water was obtained by a Milli-Q Plus system (EMD Millipore, Billerica, MA, USA). All other used chemicals were of analytical reagent grade.

### 3.3. Microwave-Assisted Extraction Protocol

The MAE was performed in mono-mode at fixed frequency. The microwave oven (MM817ASM, Bosch, Germany) was set in a homemade MAE device with a suitable glass apparatus with round bottom flask (500 mL) and cooler [23]. The kinetics of the process were determined by measuring the total extraction yield (Y), total phenolic content (TP), and total flavonoid content (TF) at specific extraction times and different irradiation powers (90, 180, 360, 600, and 800 W). Extractions were performed using 60% ethanol as the extraction solvent, and a solid–liquid ratio of 1:10 (*w*/*v*). Samples were collected at different extraction times (1, 2, 3, 4, 5, 7.5, 10, 15, 20, 25, and 30 min). The flasks were always placed at the same position of the microwave extractor without additional stirring. The crude extracts were immediately filtered through filter paper (4–12 μm pore size, Schleicher & Schuell, Germany) under vacuum (V-700, Büchi, Switzerland) immediately after extraction. The extracts were collected in glass vials and stored at 4 °C until further analysis.

### 3.4. Total Extraction Yield (Y)

Crude liquid extract (10 mL) was vacuum-evaporated and further dried to determine the total extraction yield (Y). The results were expressed as the percentage of total extractable solids per 100 g of dry peppermint leaves (%, *w*/*w*).

### 3.5. Total Phenolic Content (TP)

The TP was determined using the Folin–Ciocalteu assay [41,42]. Gallic acid was used as an analytical standard to obtain a calibration curve, and the absorbances of the samples were measured at 750 nm (6300 Spectrophotometer, Jenway, Stone, UK). The TP was expressed as mg of gallic acid equivalents (GAE) per 100 g dry weight (DW) and mg GAE per mL. Results were expressed as mean values, and all analyses were performed in triplicate.

### 3.6. Total Flavonoid Content (TF)

Total flavonoid content was determined by colorimetric assay using aluminum chloride and sodium nitrite [43]. Catechin was used as analytical standard to obtain a calibration curve, and absorbance was measured at 510 nm. Results were expressed as mg catechin equivalents (CE) per 100 g DW and mg CE/mL, and as mean values, while all analyses were performed in triplicates.

### 3.7. Mathematical Modeling

The kinetics of MAE were described mathematically by four empirical models commonly used for modeling different extraction processes. The values for Y, TP, and TF were studied, with extraction kinetics measured as function of extraction time. Experiments were also performed as a function of microwave irradiation power (90–800 W).

Variables *C_t_* (mg/mL) and *t* (min) are dependent and independent variables obtained from experimental data, while all other parameters in mathematical models were determined using the least square method. In this method, the parameters of the model are adjusted in order to minimize the sum of the squares of the differences between the predicted values of the model and the actual data points. The Levenberg–Marquardt algorithm was used in order to minimize the error function and nonlinear regression models were introduced to capture more complex relationships between variables. The parameters in nonlinear equations were obtained without linearization to avoid inaccurate predictions and biased estimates of the parameters. Each presented model is accompanied by an explanation of all its parameters and their corresponding physical meanings.

The first-order rate law model (Model I) which represents a specific solution of Fick’s law was applied first:

(1) $$C_{t}=C_{eq}\left(1-e^{-k_{1}t}\right)\;$$

where *C_t_* (mg/mL) is the concentration of the response (Y, TP, or TF) at time (*t*), *C_eq_* (mg/mL) represents it concentration at equilibrium, and *k*_1_ (min^−1^) is the rate constant. *C_eq_* and *k*_1_ were adjustable parameters in this model.

Second-order kinetics model have already been successfully applied for the modeling of MAE processes for various bioactive compounds such as camptothecin and flavonoids [44]. This model could be presented by following equation:

(2) $$\frac{dC_{t}}{dt}=k{\left(C_{eq}-C_{t}\right)}^{2}$$

where *k* (min^−1^) is the second-order rate constant. Equation (2) could be solved with the following boundary conditions: $C_{t|t=0}=0$ and $C_{t|t=t}=C_{t}$:

(3) $$C_{t}=\frac{C_{eq}^{2}k_{2}t}{1+C_{eq}k_{2}t}$$

Furthermore, Equation (3) could be reordered to obtain the final form of the Model II equation:

(4) $$C_{t}=\frac{C_{eq}t}{k_{2}^{\prime }+t}\;$$

where $k_{2}^{\prime }=\frac{1}{C_{eq}k_{2}}$.

Equation (4) also represents the often-used Peleg’s hyperbolic model obtained to describe moisture sorption kinetic curves [45]. It has been suggested that Peleg’s equation could be successfully used in order to describe extraction of different bioactive compounds from natural resources due to similarity in the shapes of extraction and sorption curves [46]. *C_eq_* and $k_{2}^{\prime }$ were adjustable parameters.

Model III applied in this work represents the Elovich’s equation which was established to describe adsorption curves [46]:

(5) $$C_{t}=E\;ln\left(t\right)+a$$

where all symbols describe adjustable parameters, and *E* represents the extraction rate constant. The equation was derived under the assumption that the leaching rate decreases exponentially with an increase in extraction yield [8].

Model IV was based on the power-law model, also commonly used for MAE of various plant materials [46,47]. The Model IV equation could be expressed as follows:

(6) $$C_{t}=Bt^{n}$$

where *n* is the power-law exponent (<1) and *B* is the constant related to the extraction rate.

### 3.8. Gas Chromatography Mass Spectrometry (GC-MS) Analysis

*GC-MS system:* Analyses of volatile compounds were conducted using an Agilent gas chromatograph-mass spectrometer equipped with a Headspace Sampler previously described by Pavlić et al. [23]. The system consists of an Agilent 7697A Headspace Sampler, 6890N Gas Chromatograph (GC) and a 5975C Mass Selective Detector. Chromatographic separation was performed on a DB-5MS (60 m × 0.25 mm ID, 0.25 µm) capillary column [23]. After the vials had been pressurized with carrier gas (helium at 103.4 kPa) for 15 min, the sampling valve with a 1 mL sample loop was used to maintain uniform sample volumes for GC-headspace analysis of volatiles in peppermint leaves extract. The loop was filled with headspace vapors of peppermint leaves extract obtained by MAE in split mode (16.2:1) for 0.5 min with a 110 °C oven temperature. The transfer line was set at 150 °C. Helium was used as the gas carrier with a constant flow mode of 1 mL/min. The initial temperature of the oven was held at 50 °C for 2 min, increased to 300 °C at a 10 °C/min, and finally kept at 300 °C for 5 min [48]. Electron ionization mode (70 eV) was set on the mass spectrometer in the mass scan range of *m*/*z* 45–550. The temperature of the ion source was set at 300 °C.

*Sample introduction:* A 2 mL aliquot of each sample extract spiked with 1-bromo-2-fluorobenzene as internal standard at a final concentration of 1.25 mg/L were placed into 20 mL headspace vials. The vials were further sealed with PTFE-lined silicone septa [23].

*Identification and quantification of compounds*: Volatile compounds released from the sample extract were tentatively identified by comparing their spectra with the reference mass spectra libraries (NIST98 and Wiley7N) and by comparing their GC Kovats indices, which were calculated using the retention times of *n*-alkanes C7–C25 (Sigma-Aldrich, St. Louis, MI, USA). Non-isothermal linear retention indices (Kovats type) were calculated after the analysis of *n*-alkane under the same conditions [23]. Semi-quantitative GC/MS analysis of volatiles in *M. piperita* extracts was achieved using 1-bromo-2-fluorobenzene as an internal standard, and results were expressed as mg per 100 g of peppermint leaves (mg/100 g).

### 3.9. Statistical Analysis

The accordance between experimentally obtained values of investigated responses (Y, TP, and TF) and predicted values were established by the sum of squared errors (SS_er_), coefficient of determination (*R*^2^), and average absolute relative deviation (AARD). Better accordance between experimental data and model was achieved when SS_er_ and AARD were minimal, while *R*^2^ was higher [49].

## 4. Conclusions

Polyphenolic compounds from organic *Mentha piperita* leaves were successfully isolated by MAE. The influences of irradiation power on Y, TP, and TF were evaluated, and the highest values obtained within the experimental domain were 40.17%, 10.17 g GAE/100 g, and 10.55 g CE/100 g, respectively. Fitting of kinetic curves for Y and TP as dependent variables were performed using four commonly used empirical models for MAE modeling. All applied models adequately fitted the experimental data in terms of the fitting quality parameters (SS_er_, *R*^2^, and AARD). The best fit was obtained by the first-order model (Model I) due to the lowest SS_er_ and AARD, and the highest *R*^2^ in the case of Y and TP. Since Model I provided the best fit, influences of irradiation power on the extraction rate constant (*k*_1_) and asymptotic yield (*C_eq_*) were determined. It was observed that that irradiation power exerted a significant influence on *k*_1_, while its influence on the asymptotic value of the response could be neglected. This is due to the absence of significant differences in yield at the end of the process (30 min). Furthermore, the highest experimentally determined *k*_1_ was obtained at an irradiation power of 600 W, while the optimal irradiation power obtained by the maximum determination of the fitting curve predicted the highest *k*_1_ (2.36 min^−1^) at 665 W. The chemical profiles of peppermint volatiles in the liquid extracts were determined by GC-MS. Here menthomenthene, menthone, and 1,8-cineole were the most abundant compounds. Further studies should be conducted to optimize MAE of the same plant material by experimental design to evaluate effects of other extraction parameters, such as solid-to-liquid ratio, solvent concentration, extraction time at fixed irradiation power, and chemical characterization of both polyphenolic and volatile fractions. There is strong scientific evidence supporting the efficiency of MAE for production of high-quality extracts due to improved extraction rates. This will enable sustainable production of value-added peppermint-based products, designed for high-value applications in the pharmaceutical and/or cosmetic and/or food sectors.

## Figures and Tables

**Table 1:** Calculated parameters and statistical data of four empirical models applied for MAE kinetics of total extraction yield.

Model Equation	P [W]	Model Parameters						Statistical Parameters		
		*k*	*C_eq_*	*E*	*a*	*B*	*n*	*R* ^2^	SS_er_	AARD [%]
$C_{t}=C_{eq}\left(1-e^{-k_{1}t}\right)$	90	0.419	37.593					0.990	15.182	4.04
	180	0.750	38.122					0.993	9.329	2.19
	360	1.187	37.152					0.992	10.466	2.27
	600	2.431	36.755					0.990	12.237	2.50
	800	1.680	38.363					0.996	5.862	1.61
Mean								0.992	10.615	2.52
$C_{t}=\frac{C_{eq}t}{k_{2}^{\prime }+t}$	90	1.747	42.109					0.974	39.715	6.33
	180	0.800	41.107					0.982	24.954	4.06
	360	0.415	39.088					0.986	17.957	3.26
	600	0.134	37.657					0.996	4.873	1.83
	800	0.233	39.685					0.997	3.623	1.27
Mean								0.987	18.224	3.35
$C_{t}=E\;ln\left(t\right)+a$	90			7.225	17.882			0.835	105.918	11.05
	180			4.701	26.218			0.730	84.126	8.14
	360			2.773	30.470			0.616	49.360	5.08
	600			1.204	34.209			0.740	5.225	1.68
	800			1.855	34.109			0.722	13.591	2.60
Mean								0.729	51.644	5.71
$C_{t}=Bt^{n}$	90					20.636	0.213	0.897	156.243	13.75
	180					27.416	0.126	0.929	100.420	8.92
	360					30.928	0.075	0.958	53.332	5.26
	600					34.273	0.033	0.996	5.392	1.72
	800					34.282	0.048	0.989	14.458	2.63
Mean								0.954	65.969	6.46

**Table 2:** Calculated parameters and statistical data of four empirical models applied for MAE kinetics of total phenols content.

Model Equation	P [W]	Model Parameters						Statistical Parameters		
		*k*	*C_eq_*	*E*	*a*	*B*	*n*	*R* ^2^	SS_er_	AARD [%]
$C_{t}=C_{eq}\left(1-e^{-k_{1}t}\right)$	90	0.403	9.236					0.984	1.461	4.90
	180	0.618	9.466					0.986	1.327	4.40
	360	1.060	9.356					0.987	4.959	2.86
	600	2.284	8.744					0.976	1.706	4.08
	800	2.164	9.202					0.985	1.161	2.95
Mean								0.984	2.123	3.84
$C_{t}=\frac{C_{eq}t}{k_{2}^{\prime }+t}\;$	90	1.764	10.307					0.984	1.395	4.67
	180	1.072	10.357					0.959	3.950	8.25
	360	0.484	9.892					0.987	5.112	3.03
	600	0.156	8.991					0.983	1.180	3.38
	800	0.149	9.430					0.988	0.919	2.86
Mean								0.980	2.511	4.44
$C_{t}=E\;ln\left(t\right)+a$	90			1.743	4.425			0.891	3.811	7.03
	180			1.415	5.797			0.672	10.033	12.94
	360			0.778	7.452			0.643	23.178	6.00
	600			0.354	8.000			0.560	1.016	2.47
	800			0.297	8.542			0.433	1.190	3.39
Mean								0.640	7.846	6.37
$C_{t}=Bt^{n}$	90					5.028	0.215	0.931	6.039	9.73
	180					6.276	0.153	0.871	12.194	13.92
	360					7.600	0.083	0.953	98.974	6.22
	600					8.017	0.041	0.985	1.019	2.48
	800					8.562	0.032	0.984	1.208	3.42
Mean								0.945	23.887	7.15

## Data Availability

Not applicable.
